# Protracted COVID-19 during Treatment of Facial Palsy

**DOI:** 10.1155/2021/5569841

**Published:** 2021-06-04

**Authors:** Mehrdad Hasibi, Maral Seyed Ahadi, Hamed Abdollahi, Mehrdad Jafari

**Affiliations:** ^1^Professor of Infectious Diseases, Amiralam Hospital, Tehran University of Medical Sciences, South Saadi St., Tehran, Iran; ^2^Assistant Professor of Neurology, Amiralam Hospita, Iranian Center of Neurological Research, Neuroscience Institute, Tehran University of Medical Sciences, South Saadi St., Tehran, Iran; ^3^Assistant Professor, Department of Anesthesiology, Amiralam Hospital, Tehran University of Medical Sciences, South Saadi St., Tehran, Iran; ^4^Assistant Professor, Department of Otolaryngology—Head and Neck Surgery, Imam Khomeini Hospital Complex, Tehran University of Medical Sciences, Valiasr hospital, Baqerkhan St., Tehran, Iran

## Abstract

Neurologic manifestations are increasingly reported as the coronavirus disease 2019 (COVID-19) pandemic continues. This is a report of a COVID-19 patient with Bell's palsy. Case Summary. A 52-year-old man with fever and malaise was tested positive for COVID-19. After a week, he developed right-sided peripheral facial palsy and was treated with corticosteroids in conjunction with antiviral treatment which resulted in complete recovery. Discussion. Concomitant treatment of corticosteroids and antiviral treatment can decrease morbidity in patients with COVID-19-related Bell's palsy.

## 1. Introduction

The COVID-19 pandemic has been a major health issue affecting millions of people around the world. Although respiratory symptoms are the main clinical features, associated neurologic manifestations are increasingly recognized. Studies have been published describing diverse neurological manifestations from anosmia to life-threatening acute necrotizing encephalopathy. Peripheral facial palsy is one of the neurologic manifestations reported with COVID-19. There is not a consensus regarding facial palsy treatment during the COVID-19 era. This is a report of the successful treatment of a patient with COVID-19-associated facial palsy.

## 2. Case Report

**Figure 1:**
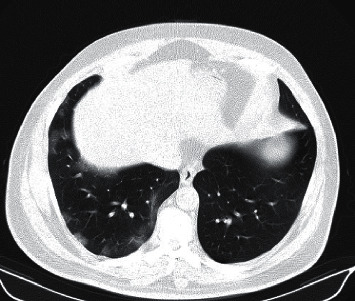
Spiral chest CT scan of the patient on day 12.

On July 10, 2020, a 52-year-old man presented to our outpatient clinic in Amiralam Hospital, Tehran, with fever, malaise, and anorexia from 2 days before without any respiratory symptoms. His blood oxygen (O2) saturation was 95% while breathing room air. Other physical examinations were unremarkable. He was diagnosed with COVID-19 based on a positive RT-PCR test result of the nasopharyngeal sample. The patient did not have any risk factors except class 1 obesity; therefore, he was managed in an outpatient setting with capsule Arbidol 200 mg (an antiviral agent) TID and naproxen 250 mg BID for 7 days. Three days later, his fever subsided and other symptoms partially resolved; however, he complained from new-onset mild dry coughs. One week following symptom onset, the patient developed right-sided facial palsy. On physical examinations, he had a grade V House–Brackmann [1] lower motor neuron facial palsy without any other cranial nerve involvement, parotid swelling, or auditory canal lesions. Other neurologic examinations were unremarkable. The result of brain MRI without contrast was normal. He was initially put on 60 mg prednisolone (to be tapered from the sixth day of treatment) and favipiravir tablet 600 mg three times a day. After 5 days of treatment, the facial palsy recovered partially but he complained of increased dry cough and severe malaise. He underwent a spiral chest CT scan which showed multiple bilateral peripheral ground glass opacities with approximately 20% involvement of the lungs (Figure 1). He was admitted and received remdesivir (200 mg on the first day and continued with 100 mg daily from the 2^nd^ to 5^th^ day, afterwards). Oral prednisolone was changed to IV dexamethasone 4 mg BID. The treatment was continued for 5 days. The patient was discharged on the sixth day of admission without any antiviral agents or oral prednisolone tapering. On the discharge day, his facial palsy improved to the grade II/VI and he completely recovered from COVID-19 respiratory symptoms. After three months of follow-up, facial palsy improved completely. Written consent was obtained from the patient for reporting and publishing the results.

## 3. Discussion

This is a report of unilateral peripheral facial palsy in a COVID-19 patient with an excellent response to combination therapy of corticosteroids and remdesivir. Since the start of the pandemic, various antivirals have been administered off label with the succeeding articles justifying their use. A recent study by Beigel et al. has shown that remdesivir reduces the time to recovery in hospitalized COVID-19 patients [2]. Other studies have shown favapiravir to be effective as an oral agent in reducing viral clearance time [3, 4].

Bell's palsy has an annual incidence of 11.5–53.3 cases per 100,000 in a year in different populations [5]. However, there are increasing reports of Bell's palsy during the pandemic, either due to the viral infection or vaccination [6]. In a study by Codeluppi et al., there was an increased incidence of facial palsy during the COVID-19 pandemic and 21% of patients with Bell's palsy had a recent and active infection [7]. Prior evidence indicates that corticosteroids are effective in the treatment of Bell's palsy. However, there are debates on corticosteroid therapy during the COVID-19 pandemic, and it is reserved for serious cases. A recent consensus recommends treatment with short courses of corticosteroid therapy in grade V and VI Bell's palsy, in the absence of signs and symptoms suggestive of COVID-19 [8]. The concern about corticosteroid therapy is more in patients with COVID-19. Although corticosteroids are one of the mainstays of treatment in severe and critical COVID-19, it is not recommended in patients with mild and moderate symptoms due to potential disease exacerbation [9, 10]. It seems that facial palsy associated with COVID-19 should be treated based on each patient's medical condition, as worse motor function based on House–Brackmann decreases the probability of complete recovery [11, 12]. Goh et al. reported a case of facial palsy 6 days after disease onset. The patient had an isolated lower motor neuron left facial palsy, and he was treated with oral prednisone and valacyclovir and lopinavir/ritonavir to reduce viral replication. However, he did not report a significant improvement after a week of treatment [13]. In another case report, a 57-year-old woman developed an acute left-side facial palsy, a week after developing symptoms of COVID-19. She only received symptomatic treatment and was reported to have a complete recovery after a month [14]. Figueiredo et al. reported a pregnant woman presented with an isolated facial palsy. She had a positive RT-PCR test for SARS-Cov-2 and was treated with corticosteroid, but the outcome was not reported [15]. In a case series by Lima et al., eight patients with COVID-19 and Bell's palsy were evaluated for clinical presentation and outcome [16]. Their patients had mild respiratory and systemic manifestations and grade II-III House–Brackmann, and none required hospitalization; seven patients received steroids from which four patients had complete recovery and three patients had partial recovery. However, concomitant use of antiviral agents is not reported.

There are reports of Bell's palsy following COVID-19 vaccination. Repajic et al. reported the first case of Bell's palsy following the 2nd dose of Pfizer-BioNTech COVID-19 vaccination in a 57-year-old Caucasian female. She was treated with prednisone and an antiviral agent, and her symptoms started to improve from the 14^th^ day [17]. Their choice of antiviral treatment is not reported. Colella et al. also reported a patient developing Bell's palsy after the first dose of Pfizer-BioNTech COVID-19 vaccination [6]. He had a grade V House–Brackmann and received corticosteroids without improvement after 23 days of follow-up.

In our patient, soon after starting corticosteroid treatment, the course of COVID-19 progressed despite oral favipiravir therapy. In our experience, short-term corticosteroid therapy in combination with remdesivir may prove beneficial in patients with severe facial palsy associated with COVID-19. We suggest conduction of future clinical trials on concomitant administration of a potent antiviral agent and corticosteroid therapy in patients with severe Bell's palsy associated with COVID-19 to prevent potential disease exacerbation.

## Data Availability

Data are available upon request from the authors.
